# Cellular and molecular signatures of motherhood in the adult and ageing rat brain

**DOI:** 10.1098/rsob.230217

**Published:** 2023-11-22

**Authors:** P. Duarte-Guterman, J. E. Richard, S. E. Lieblich, R. S. Eid, Y. Lamers, L. A. M. Galea

**Affiliations:** ^1^ Department of Psychology, University of British Columbia, Vancouver, British Columbia, Canada; ^2^ Graduate Program in Neuroscience, University of British Columbia, Vancouver, British Columbia, Canada; ^3^ Djavad Mowafaghian Centre for Brain health, University of British Columbia, Vancouver, British Columbia, Canada; ^4^ Institute for Neuroscience and Physiology, University of Gothenburg, Sweden

**Keywords:** parity, neuroplasticity, postpartum, neuroinflammation, middle age, tryptophan–kynurenine

## Abstract

Pregnancy is marked by robust changes, including brain changes to volume, structure, connectivity and neuroplasticity. Although some brain changes are restricted to pregnancy and the postpartum, others are long-lasting. Few studies have examined possible mechanisms of these changes or the effects of multiple pregnancies. We characterized various cellular and molecular signatures of parity (nulliparous, primiparous, biparous) in the rat hippocampus. We investigated density of neural stems cells (Sox2), microglia (Iba-1) and levels of a synaptic protein (PSD-95), cell signalling pathways, neuroinflammation, and the tryptophan–kynurenine (TRP–KYN) pathway, one week after weaning their pups from the last pregnancy (age of dam: seven months) and in middle-age (age of dam: 13 months). Parity increased PSD-95 levels in both age groups and prevented the age-related decrease in neural stem cell density observed in nulliparous rats. Biparity increased cell signalling phosphoproteins (pp70S6K, S6RP) and number of microglia in the dentate gyrus, regardless of age. Parity resulted in transient changes to the TRP–KYN system. Thus, previous parity has lasting effects on synaptic plasticity with fewer lasting effects on inflammation and cell signalling phosphoproteins in the whole hippocampus.

## Graphical abstract

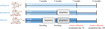

## Highlights

— Parity increased the postsynaptic protein PSD-95 in the hippocampus, regardless of age.
— Biparity increased microglial density in the hippocampus, regardless of age.
— Parity prevented the age-related decline in hippocampal neural stem cells.
— Parity transiently increased tryptophan–kynurenine pathway metabolites.
— Parity did not substantially alter plasma or hippocampal cytokine levels, regardless of age.

## 1. Introduction

During pregnancy, the maternal brain undergoes substantial remodelling which can lead to long-lasting effects on brain physiology [1,2] (reviewed in [3,4]). In primigravid individuals, i.e. individuals who are pregnant for the first time, total brain volume and grey matter are reduced at parturition, but rebound approximately six months postpartum [5–11]. Multiple brain regions are remodelled during pregnancy with some areas recovering [5,6,12,13] but others, such as regions within the prefrontal cortex, showing enduring changes, present years later [2].

The hippocampus, which is known for its role in spatial learning and memory, undergoes profound functional and structural changes during gestation and postpartum and plays an important role for motherhood [14]. Hippocampal volume is reduced after giving birth and does not fully recover within 6 years after parturition [2,15]. The hippocampus is unique as it shows a remarkable amount of plasticity throughout life [16–18]. An important consideration is that a reduction in volume need not be associated with impaired function, but instead could indicate fine tuning of the maternal brain to the demands of motherhood [2,4,19,20], and work in animal models indicates a facilitation of hippocampus-related cognition after the pups have weaned [21]. Furthermore, the hippocampus plays an important role in maternal caring behaviours, for instance nest building and pup retrieval in rodents [22,23].

Although most studies focus on the short-term effects of pregnancy on the brain, long-lasting signatures of previous pregnancy have been reported in the ageing brain. In middle-aged and older-aged women, previous parity is associated with less evident brain ageing, including in the hippocampus [24,25]. In rats, adult hippocampal plasticity changes both in the short- and long-term following pregnancy [1,26–30]. Previous parity may also alter the risk of neurological diseases later in life, for instance Alzheimer's disease [31], and alter the rate of disease progression [32,33]. Although these studies show long-lasting effects of parity in the brain, few studies have examined the cellular and molecular signatures that may act as possible mechanisms of these changes.

Microglia, the resident immune cells in the brain, regulate many aspects of neuronal physiology, including neurogenesis, synaptogenesis and synaptic pruning [34–37]. The neuroimmune system adapts during pregnancy and in the postpartum [34,38–42], but few studies have investigated if these changes persist long term ([1,26]; reviewed in [43]). In the hippocampus, the number of microglia are reduced and levels of certain cytokines (interleukin (IL)-6 [1,34,42,44] and IL-10 [45]) are increased during pregnancy and early postpartum], with some changes in microglia and peripheral cytokines persisting long term [1]. Pregnancy-induced changes to neuroimmune signalling may therefore cause long-lasting changes to hippocampal function, although little is known about how these transient changes in immune signalling affect brain health and processing beyond pregnancy and postpartum. Furthermore, little is known about the short- and long-term neuroimmune consequences of increasing amount of parity.

Pregnancy also alters the tryptophan (TRP)–kynurenine (KYN) pathway [46], which has a direct relationship with the inflammatory system [47]. The TRP–KYN pathway also regulates hippocampal neurogenesis and inflammation, and the KYN pathway is altered with age, and alterations in its activity are associated with a variety of age-associated neurological diseases [48]. To our knowledge, no study has investigated the longer-term effects of pregnancy on the TRP–KYN pathway.

Our objective was to perform a wide-ranging study of the cellular and molecular signatures of parity in the hippocampus after dams had weaned their pups, and in middle age, to elucidate the specific effects of parity on brain health. This investigation will not only shed light on the unique alterations induced by reproductive history but also facilitate a deeper understanding of how these changes influence disease susceptibility and the overall trajectory of ageing-related disorders. Therefore, we investigated the effect of nulliparity, primiparity and biparity on synaptic protein post-synapse density (PSD-95), ribosomal phosphoprotein S6 kinase (pp70S6K) and S6 ribosomal protein (S6RP), neuroinflammation markers (cytokines, chemokines), TRP–KYN pathway metabolites, and cell signalling proteins of the ERK and Akt pathways in the hippocampus. We also examined hippocampal neural stem cells (Sox2 + cells) and microglia (Iba-1). We expected that parity would increase neuroinflammation and reduce neuroplasticity in the hippocampus in the short term, and that these factors would normalize or reverse long term compared to nulliparous females, dependent on the amount of parity.

## 2. Material and methods

### 2.1. Animals

Adult male and female Sprague-Dawley rats (two months old) were obtained from Charles River (Quebec, Canada). Male rats were only used for breeding purposes. In total, 67 female rats were included in the study, with 9–10 females per parity group per age. Rats were given ad libitum access to food and water and double-housed, except during gestation and in the postpartum until weaning in which they were single-housed (from pregnancy confirmation until after weaning at 23 days postpartum). Nulliparous rats were pair-housed in a separate colony room to prevent nulliparous females from being exposed to males and to offspring but were single-housed for a period equivalent to parous groups (length of time from pregnancy to weaning). For the long-term groups, females were single housed until 45 days after giving birth. At that point, females were pair-housed until tissue and sample collection. All protocols were approved by the Institutional Animal Care Committee at the University of British Columbia and conformed to the guidelines set out by the Canadian Counsel on Animal Care.

### 2.2. Breeding and maternal observations

**Figure 1.**
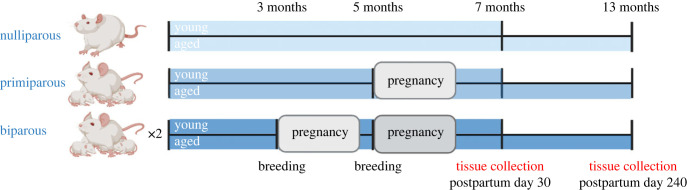
Graphical representation of experimental timeline, including pregnancies and tissue/sample collection. Females were divided into three groups, in which one group did not partake in any breeding (nulliparous), one group was bred at five months and gave birth to one litter (primiparous), one group was bred at three and five months and gave birth to two litters (biparous). Parity groups were further divided into two, in which the first group was euthanized 30 days postpartum (young) and the second group was aged until 13 months (postpartum day 240) before euthanasia and tissue collection. Litters were culled 1 day postpartum (four female and four male offspring per dam) and offspring were weaned 23 days after birth. *n* = 9–11 per group. Image created using BioRender.com.

For breeding, two females and one male were paired overnight. Females paired with males were vaginally lavaged each morning, and samples were assessed for the presence of sperm. If sperm was present, the female was considered pregnant and was single-housed into a clean cage. Pregnant females were monitored weekly throughout gestation (weighed and abnormalities noted). One day after birth (postpartum day 1), litters were culled to four males and four females. Original litter sizes are presented in electronic supplementary material, table S1. Maternal behaviour (time spent licking, nursing, off-nest) was scored between postnatal days 2–8, as previously described [49] (electronic supplementary material, table S1). One female did not have enough pups, therefore we cross-fostered with pups from other females born on the same day. All animals were age-matched such that primiparous rats were the same age as biparous rats during their second pregnancy. Biparous rats were bred at three months and five months of age and primiparous females were bred at five months of age. Timeline is illustrated in figure 1.

### 2.3. Blood and tissue collection

**Figure 2.**
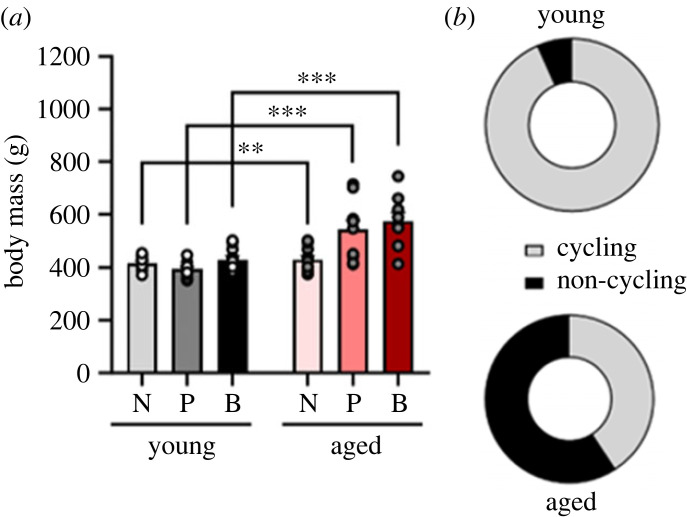
Body mass increases in middle-aged regardless of parity. Female rats displayed increased body mass in all parity groups (*a*). Proportion of cycling and non-cycling females in each age group are presented in (*b*). Data are expressed as mean ± s.e.m. Body mass data: *n* = 9–10 per group. **p < 0.01, ***p < 0.001.

To monitor oestrous cycles, vaginal lavages were conducted for 6–11 days prior to euthanasia and on the day of euthanasia to determine whether cycling had restarted after pregnancy in the young adult animals or had stopped in the older animals (cycling data are reported in figure 2*c*). On postpartum day 30 (adult) and 240 (middle-aged) all rats received a lethal overdose of sodium pentobarbital between 10.00 and 12.00, to limit any circadian influence. Blood was collected via cardiac puncture into cold EDTA coated tubes and centrifuged (within 2 h from time of dissection) for 10 min at 4°C and plasma was stored at −80°C. Brains were quickly extracted, and hippocampus was rapidly dissected from the left hemisphere over ice, flash frozen on dry ice and stored at −80°C (*n* = 10 per group). For one subset of animals (*n* = 9–10 per group), the right hemisphere (without olfactory bulbs and cerebellum) was flash frozen on dry ice for subsequent brain punches and analysed using electrochemiluminescence immunoassay kits. For the other subset of animals (*n* = 3–6 per group), the right hemisphere was fixed for 24 h in 4% paraformaldehyde in phosphate-buffered saline (PBS; 4°C) and then transferred to a 30% sucrose/PBS solution for cryoprotection. Serial coronal sections (30 µm) were cut with a freezing microtome (SM2000R; Leica, Richmond Hill, Ontario, Canada) across the extent of the hippocampus (collected in 10 series) and stored in an antifreeze solution (containing 0.1 M PBS, 30% ethylene glycol and 20% glycerol) and stored at −20°C for immunohistochemistry.

### 2.4. Plasma TRP–KYN metabolite quantification

We quantified KYN, kynurenic acid (KA), xanthurenic acid (XA), anthranilic acid (AA), 3-hydroxykynurenine (3-HK), 3-hydroxyanthranilic acid (3-HAA), riboflavin (vitamin B2) and its cofactors, flavin adenine dinucleotide (FAD), flavin mononucleotide (FMN), pyridoxine (vitamin B6), pyridoxic acid, pyridoxal, pyridoxal 5′-phosphate and pyridoxamine from plasma, using isotope dilution liquid chromatography coupled with tandem mass spectrometry (ABSciex API4000; AB SCIEX, Framingham, MA, USA) based on a modified method by Midttun *et al*. [50]. Neuroprotective-to-neurotoxic *z*-score ratios were calculated to account for the balance between neuroprotective metabolites or neurotoxic metabolites/cofactors: zKA/zHA and zKA/zHK. A similar approach was used in [51].

### 2.5. Hippocampal PSD-95, cytokines and cell signalling levels

Electrochemiluminescence immunoassay kits (Meso-Scale Discovery; Rockville, MD) were used to measure PSD-95 (cat. no. K150QND), ERK-1/2, JNK, p38 (MAP Kinase Whole Cell Lysate Kit; cat. no. K15101D), Akt, GSK-3β, pp70S6K, S6RP (Akt Signalling Panel II Whole Cell Lysate Kit; cat. no. K15177D) and cytokines (IFN-γ, IL-1β, IL-4, IL-5, IL-6, IL-10, IL-13, CXCL-1 and TNF-α; V-PLEX Proinflammatory Panel 2 Rat Kit; cat. no. K15059D) in homogenized whole hippocampal samples. Levels of K15116, MEK 1 and 2, and STAT3 were also measured, although levels were undetectable. All Meso-scale Discovery kits were developed for use in mouse, rat and human lysates, except for the proinflammatory panel which is specific to rat tissues. Hippocampi were homogenized using a bead disruptor (Omni, VWR) in 200 µl of cold Tris lysis buffer (150 mM NaCl, 20 mM Tris, pH 7.5, 1 mM EDTA, 1 mM EGTA, 1% Triton X) containing a cocktail of protease and phosphatase inhibitors. Homogenates were centrifuged at 2100*g* for 10 min at 4°C and supernatants were aliquoted and stored at −80°C. Protein concentrations were determined using a BCA protein assay kit (ThermoFisher). Homogenates were diluted with Tris lysis buffer 1 : 10 for PSD-95 and Akt kit plates and 1 : 100 for MAP kinase kit. Samples were run in duplicate following the manufacturer's protocols except that plates were incubated overnight at 4°C (instead of 3 h). Plates were read with a MESO QuickPlex SQ 120 (Meso Scale Discovery) and data were analysed using the Discovery Workbench 4.0 software (Meso Scale Discovery). Cytokine levels were obtained using a standard curve and expressed as pg/mg protein. For PSD-95 and cell signalling assays, background signal was subtracted from raw signal and normalized to protein levels and results are expressed as signal/mg ml^−1^ protein. Cytokines were not measured in three females (one primiparous aged female and two biparous aged) due to the presence of hock sores or cysts/tumors (ovarian or mammary gland) found at the time of dissection.

### 2.6. Immunohistochemistry

A subset of four to six female brains per parity and age group were used for immunohistochemistry staining and Iba1 and Sox2 quantification and analysis. A series of free-floating tissue was rinsed 3× with 0.1 M PBS (Iba1 stain; pH 7.4) or tris-buffered saline (TBS; Sox2 stain; pH 7.4), on a rotator, for 10 min each at room temperature (RT). Tissue was then incubated in 3% hydrogen peroxide for 30 min at RT. Sections were added to the blocking solution (Iba1: 10% normal goat serum [NGS] and 0.4% Triton X 100 in PBS, for 1 h; Sox2: 3% normal horse serum [NHS] and 0.3% Triton X 100; for 30 min). Tissue was immediately incubated in a primary antibody solution (Iba1: 1 : 1000 rabbit anti-Iba1 [Wako Chemicals USA] in PBS containing 5% NGS, 0.4% Triton X, for approximately 20 h at 4°C; Sox2: mouse anti sox2 monoclonal; 1 : 1000 [Santa cruz Biotechnology, sc-365 964] in TBS containing 3% NHS, 0.4% Triton X, for approximately 40 h at 4°C). Following PBS rinses, tissue was incubated (Iba1: in a biotinylated goat anti-rabbit antibody [1 : 500; Vector laboratories] in PBS containing 2.5% NGS, 0.4% Triton-X, for 1.5 h; Sox2: ImmPRESS HRP Horse anti-Mouse IgG, [1 : 2, Rat adsorbed, Vector laboratories] diluted in TBS, for 2 h). Iba1-stained sections were then incubated in avidin-biotin horseradish peroxidase complex kit (1 : 500, Vector Laboratories) in PBS containing 0.4% Triton-X for 1 h. Tissue was incubated in 3,3′-diaminobenzidine Peroxidase Substrate Kit, Vector Laboratories using 0.175 M sodium acetate as the buffer for approximately 4 min (Iba1) or approximately 7.5 min (Sox2). Tissue was rinsed 3× quickly, then another 3 × 10 min each to stop the reaction. Finally, tissue was mounted onto Superfrost/Plus slides (Fisher Scientific), allowed to dry a few days, then dehydrated in ethanol, cleared with Xylene, and coverslipped immediately with Permount (Fisher Scientific). The primary antibody was omitted as a control and this tissue did not contain any immunoreactivity.

### 2.7. Microscopy analysis

A researcher blind to experimental conditions counted Iba1+ cells in one hemisphere for the entire rostrocaudal (dorsal and ventral) extent of the hippocampus at 400× magnification using a standard light microscope. In the GCL, counts were divided by GCL volume to obtain cell densities (counts per mm^3^). GCL volumes were quantified from digitized images using Cavelier's principle, multiplying the sum of the area of each section by the section thickness (30 µm) [52]. Additionally, we examined Iba1+ cell morphology in the GCL only as a proxy for functional state, following previously described methods [1]. Briefly, an experimenter blinded to condition categorized Iba1+ cells into ramified (highly branched with long processes), stout few (shorter processed) or ameboid microglia (no processes) in the GCL of the DG. For the CA1 and CA3 regions, photomicrographs of three dorsal and three ventral sections (six sections total per animal) were taken at 10× magnification using the same exposure and gain settings. Owing to higher Iba1+ cells in the CA1 and CA3 regions of the hippocampus we used a region of interest (0.39 mm × 0.57 mm) approach, placed to include the pyramidal and radiatum layers.Representative image of Iba1+ cells in the hippocampus demonstrated.

A researcher blind to experimental conditions counted Sox2 + cells in the SGZ and GCL. Sox2 + cells were counted in the SGZ and GCL of the dentate gyrus in five dorsal and five ventral sections (10 sections total per animal) using segmentation and object classification analysis in the density counting workflow in Ilastik [53]. Photomicrographs were taken at 4× magnification. Dorsal and ventral regions were trained separately due to differences in background. Five (dorsal) and six (ventral) images were used for training to define objects (Sox2 + cells) and background. Images for training were chosen from the two ages (7 and 13 months). Images were exported to ImageJ for tracing the GCL and SGZ regions on each section and density of Sox2 + cells were obtained using the integrative density function.Representative images of Sox2 + staining in the hippocampus are demonstrated in figure 4*a,b*.

### 2.8. Statistical analysis

All the data are presented as mean ± s.e. of the mean. Data were analysed using TIBCO Statistica software (v. 9, StatSoft, Tulsa, OK, USA) using a two-way general linear factorial ANOVA with age (young, aged) and parity (nulliparous, primiparous, biparous) as between-subjects factors for each variable. Effect sizes are reported for significant effects in which Cohen's *d* is reported for the size of the difference between two means, whereas partial eta squared ($\eta_{p}^{2}$) is reported for ANOVA main or interaction effects [54]. Post-hoc comparisons used Newman–Keul's. *A priori* we expected differences by amount of reproductive experience and ageing, and any *a priori* analyses used Bonferroni corrections. For cytokines and TRP–KYN metabolites, principal component analyses (PCA) were conducted to determine networks (components) that could explain metabolite variances. Principal component scores for individual sample data for the first three components were generated using R statistical software (v. 4.1.2) and analysed using two-way ANOVA models to derive information regarding the amount of variance accounted for by metabolite profiles. Frequency of cycling between the age groups was compared using Pearson's chi-square (*χ*^2^) test. Data are plotted using GraphPad Prism (GraphPad Software, Inc., San Diego, CA). *p*-values lower than 0.05 were considered statistically significant.

## 3. Results

### 3.1. Increased age is associated with increased body mass and reduced cycling

As expected, middle-aged females had higher body mass than young adult females (main effect of age, *F*_1, 52_ = 50.022, *p* *=* 0.000, $\eta_{p}^{2}=0.490$), regardless of parity (nulliparous versus nulliparous: *p* *=* 0.002, Cohen's *d* = 1.574; primiparous versus primiparous: *p* *=* 0.0003, Cohen's *d* = 2.168; biparous versus biparous: *p* < 0.0009, Cohen's *d* = 1.998836). There was no significant main effect of parity (*p* *=* 0.527) and no significant interaction between parity and age regarding body mass (*p* *=* 0.759; figure 2*a*).

As expected, middle-aged rats were more likely to be non-cycling than younger adult rats ($\chi_{1,57}^{2}=18.192$, *p* = 0.00002; figure 2*b*).

### 3.2. Parity increases synaptic plasticity and cell signalling phosphoproteins in the hippocampus

**Figure 3.**
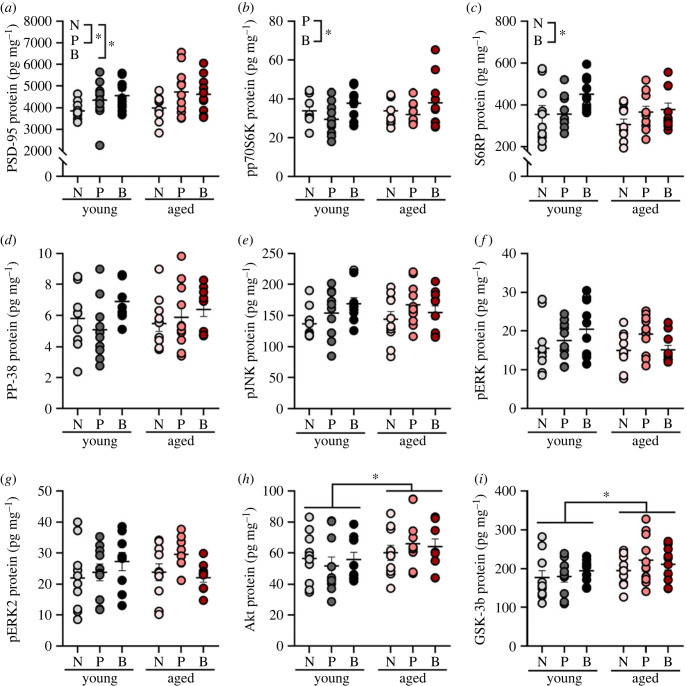
Parity increased synaptic plasticity in the hippocampus. Parity increased the synaptic protein PSD-95 (*a*) and phosphoprotein pp70S6K (*b*), and cell signalling phosphoprotein S6RP (*c*), regardless of age. Age significantly increased the levels of Akt (*h*) and GSK-3b (*i*). Parity did not significantly affect protein levels of PP-38 (*d*), pJNK (*e*), pERK (*f*), pERK2 (*g*), Akt (*h*) and GSK-3b (*i*), although age significantly increased levels of GSK-3b (*i*). Data are expressed as mean ± s.e.m. *n* = 9–11 per group. * *p* < 0.05.

We investigated the effects of parity on cell signalling and synaptic proteins in the hippocampus. Parity significantly increased PSD-95, regardless of age (main effect parity: *F*_2, 55_ = 4.612, *p* = 0.014, $\eta_{p}^{2}=0.143$; figure 3*a*), with both primiparous and biparous animals having more PSD-95 expression relative to nulliparous animals (all *p* < 0.022, Cohen's *d* ≥ 0.813). There were no other significant main or interaction effects (*p* > 0.349).

Biparity increased expression of the phosphoprotein ribosomal protein S6 kinase (pp70S6K) relative to the primiparous group (*p* = 0.020, Cohen's *d* = 0.812) but not the nulliparous group (*p* = 0.122; main effect of parity: *F*_2, 55_ = 3.952, *p* = 0.025, $\eta_{p}^{2}=0.126$; figure 3*b*). Biparity increased expression of the phosphoprotein S6RP relative to nulliparity (*p* = 0.019, Cohen's *d* = 1.063) but not primiparity (*p* *=* 0.342; main effect of parity: *F*_2, 55_ = 2.959, *p* = 0.060, $\eta_{p}^{2}=0.097$; figure 3*c*). There were no other main effects or interactions for these phosphoproteins (all *p* > 0.180).

Ageing increased the expression of the phosphoproteins Akt and GSK-3b (main effect of age: Akt *F*_1, 53_ = 4.276, *p* = 0.044, $\eta_{p}^{2}=0.075$ and GSK-3b *F*_1, 53_ = 4.658, *p* = 0.035, $\eta_{p}^{2}=0.081$; figure 3*h–i*), with higher levels of these phosphoproteins in middle age, than in young adult females (Akt: *p* = 0.040, Cohen's *d* = 54.798 and GSK-3b: *p* = 0.038, Cohen's *d* = 0.552), but there were no other significant main or interaction effects (*p* > 0.431). There were no other main effects or interactions for any of the other phosphoproteins examined (all *p* > 0.137; figure 3*d–g*).

As maternal experiences can affect synaptogenesis [55], we explored potential differences in litter size and maternal behaviour, for example, time spent nursing, time spent licking and time spent off nest. Results are presented in electronic supplementary material, table S1, in which no significant differences were found between parity and age groups.

Taken together, hippocampal PSD-95, pp70S6K and S6RP levels were increased with parity, while levels of phosphoproteins Akt and GSK-3b increased with age only.

### 3.3. Ageing reduced density of neural stem cells but less so in parous groups and biparous rats had greater density of microglia in the dentate gyrus

**Figure 4.**
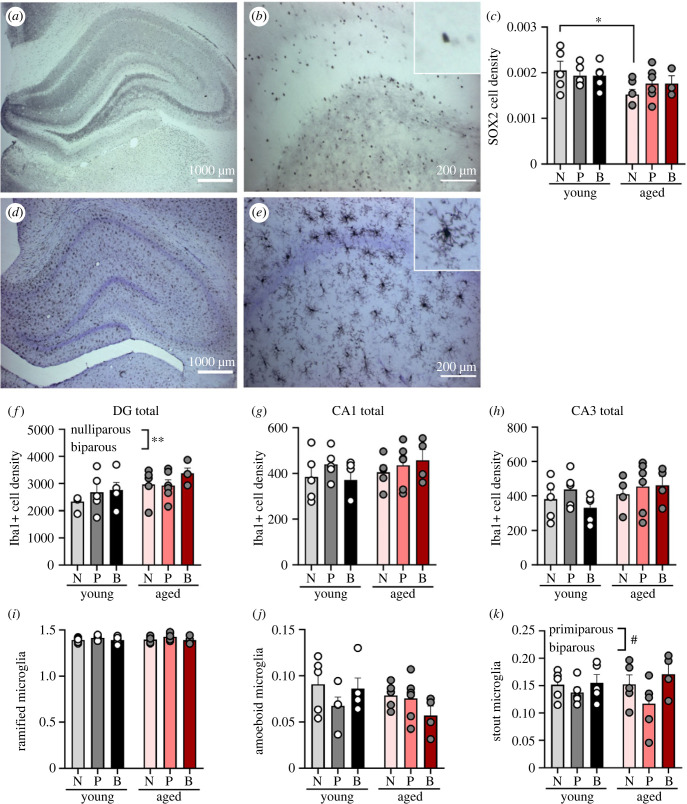
Increasing age decreases neural stem cells in nulliparous rats only, and increases microglia density in the hippocampus regardless of parity. (*a*,*b*) Representative images of SOX2 positive cells at 40× (*a*) and 200× (*b*). (*c*) SOX2 cell density is reduced with age in nulliparous, but not parous females. (*d*,*e*) Representative image of Iba1+ positive cells at 40× (*d*) and 200× (*e*). (*f*) Iba1+ cell density is significantly higher in biparous females, compared with nulliparous females, in the dentate gyrus (DG). (*g*,*h*) No differences in the Iba1+ cell density was found in the CA1 (*g*) or CA3 (*h*) regions. (*i*–*k*) Proportion of ramified (*i*), amoeboid (*j*) and stout (*k*) microglia (Iba1+ cells). Data are expressed as mean ± s.e.m. *n* = 4–6 per group for Iba1+ and 3–6 per group for SOX2. *p < 0.05, # = 0.071. N, nulliparous; P, primiparous; B, biparous.

Next, we investigated if parity alters the density of SOX2 + neural stem cells within the dentate gyrus (representative images of SOX2 + staining in figure 4*a,b*). Not surprisingly, middle age was associated with a reduction in SOX2 + cell density (main effect age: *F*_1, 24_ = 5.614, *p* = 0.026, $\eta_{p}^{2}=0.078$). However, this was driven by a larger effect size in nulliparous compared to parous groups [*a priori* comparisons adjusted using Bonferroni correction: nulliparous (*p* = 0.013, Cohen's *d* = 0.978); primiparous (*p* = 0.387, Cohen's *d* = 0.582); biparous (*p* = 0.484, Cohen's *d* = 0.584) rats] (figure 4*c*). There were no other significant effects (all *p* *>* 0.379).

Because immune processes impact hippocampal plasticity, we next investigated potential effects of age and parity on microglial number (representative image displayed in figure 4*d,e*). There was a greater density of Iba1+ cells in the dentate gyrus than any other hippocampal subregion (main effect of subregion: *F*_2, 44_ = 1026.099, *p* ≤ 0.00001, $\eta_{p}^{2}=0.979$; figure 4*f–h*). Middle-aged rats had more microglia in the dentate gyrus than younger adult rats (main effect of age: *F*_1, 24_ = 6.129, *p* = 0.021, $\eta_{p}^{2}=0.203$). Biparous rats had a greater density of Iba1+ cells in the dentate gyrus compared with nulliparous (*p* = 0.001, Cohen's *d* = 0.671) rats (*a priori* comparisons; figure 4*f*), but not primiparous rats (*p* = 0.166, Cohen's *d* = 0.373), regardless of age. There were no other significant main or interaction effects (all *p* > 0.263).

As for Iba1+ cell morphology, there was a trend towards a reduction in stout Iba1+ cells in the dentate gyrus in primiparous females, compared to biparous females (*p* = 0.071, Cohen's *d* = 1.042; main effect of parity: *F*_2, 24_ = 2.877, *p* = 0.076, $\eta_{p}^{2}=0.193$; figure 4*k*). There were no other significant effects (all *p* > 0.510). There were no significant effects for ramified or amoeboid Iba1+ cells (all *p* > 0.112; figure 4*i,j*).

Taken together, the amount of hippocampal neural stem cells decreases with age in nulliparous rats, but not parous rats (primiparous nor biparous), and microglial density in the hippocampus increases regardless of parity.

### 3.4. Minimal effects of ageing and parity on hippocampal cytokines

**Figure 5.**
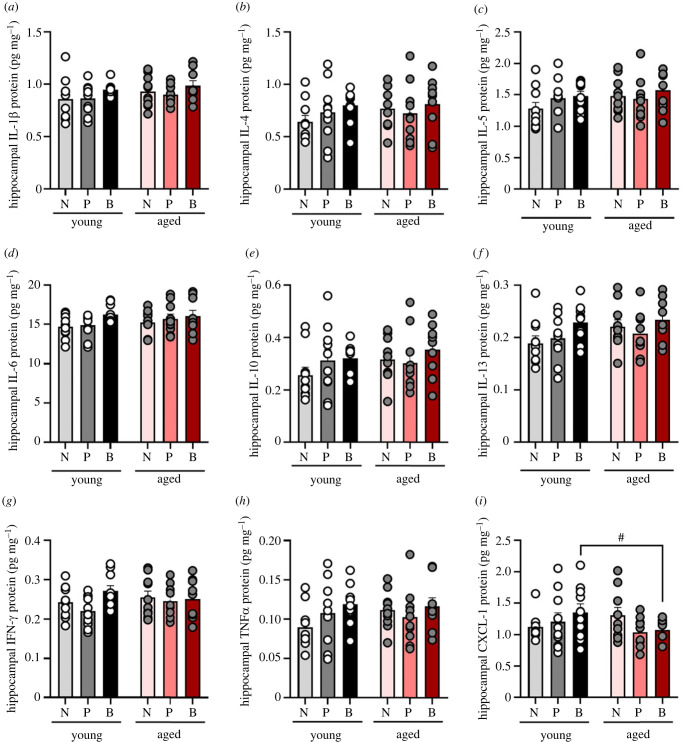
Levels of hippocampal cytokines in young adult and aged females of different parity. Protein levels of cytokines interleukin (IL)-1*α* (*a*), IL-4 (*b*), IL-5 (*c*), IL-6 (*d*), IL-10 (*e*), IL-13 (*f*), interferon (IFN)-γ (*g*), tumour necrosis factor (TNF)-α (*h*) and chemokine ligand (CXCL)-1 (*i*) measured in the hippocampus. #*p* = 0.072. Data are presented in pg/mg and expressed as mean ± s.e.m. *n* = 9–10 per group.

There was a trend towards a reduction in hippocampal CXCL-1 levels in aged compared with young biparous females (*p* = 0.072, Cohen's *d* = 0.855; interaction between age and parity: *F*_2, 52_ = 3.368, *p* = 0.083, $\eta_{p}^{2}=0.090$; figure 5*i*). There was also a trend towards a significant main effect of parity for hippocampal IL-6 (IL-6: *F*_2, 53_ = 2.463, *p* = 0.095, $\eta_{p}^{2}=0.085$; figure 5*d*). There were no significant effects for hippocampal IL-1β (*p* > 0.113, figure 5*a*), IL-4 (*p* > 0.366, figure 5*b*), IL-5 (*p* > 0.229, figure 5*c*), IL-10 (*p* > 0.299, figure 5*e*), IL-13 (*p* > 0.111, figure 5*f*), IFN-γ (*p* > 0.132, figure 5*g*) or TNF*α* (*p* > 0.221, figure 5*h*). These inflammatory factors were also measured in plasma (electronic supplementary material, figure S1). We found that plasma levels of IL-4, IL-6 and IL-10 reduced with age (main effect of age: *F*_2, 23_ = 6.547, *p* = 0.018, $\eta_{p}^{2}=0.222$ [IL-4]; *F*_1, 10_ = 4.977, *p* = 0.050, $\eta_{p}^{2}=0.332$ [IL-6]; *F*_1, 43_ = 9.093, *p* = 0.004, $\eta_{p}^{2}=0.175$ [IL-10]). There were no other significant effects (*p* > 0.247). However, many samples were under the detection limit, rendering few samples for analyses in each parity group. Therefore, we can not rule out that potential differences were missed as these values could not be included.

**Figure 6.**
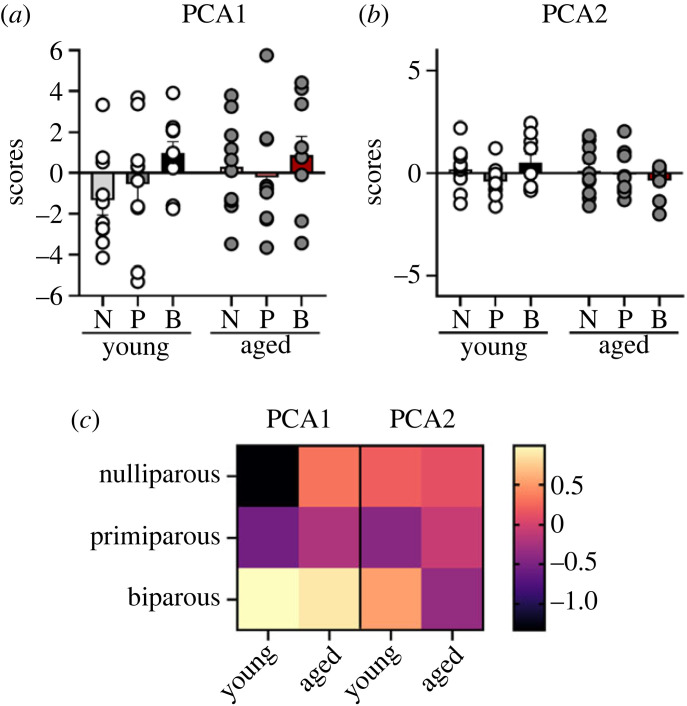
PCA for cytokines in the hippocampus. (*a*,*b*) Loadings of scores for each subject onto PCA 1 (*a*) and 2 (*b*). (*c*) Heat map displaying average loading of each parity group on each compartment. No significant differences between parity or age groups were found. Data are expressed as mean ± s.e.m. *n* = 7–9 per group.

We also analysed the hippocampal cytokines using PCA. Component loading are displayed in table 1. Results from the PCA analysis yielded two principal components, which together explained 80.98% of the total variance. All nine cytokines examined loaded positively onto PCA1 which explained 68.74% of the variance (table 1). No significant effects between parity and age groups were demonstrated in PCA1 (all *p* > 0.141; figure 6*a*).

**Table 1.** Component loadings of the hippocampal cytokine variance of components identified using PCA.

inflammatory factor	correlation	*p*-value
PCA1		
IL-13	0.9541556	1.516197 × 10^−31^
TNF-α	0.9344437	3.096247 × 10^−27^
IL-4	0.8819201	2.869053 × 10^−20^
IL-10	0.8818740	2.899291 × 10^−20^
IL-5	0.8791033	5.399171 × 10^−20^
IL-6	0.8143054	4.381999 × 10^−15^
IFN-γ	0.8046411	1.616056 × 10^−14^
IL-1β	0.7443617	1.407841 × 10^−11^
CXCL-1	0.4578589	2.661120 × 10^−4^
variance explained: 68.74%		
PCA2		
CXCL-1	0.5457298	7.82 × 10^−6^
IFN-γ	0.3938848	2.02 × 10^−3^
II-6	0.3690473	4.02 × 10^−3^
IL-1β	0.3380654	8.82 × 10^−3^
IL-4	−03296997	1.08 × 10^−2^
IL-5	−0.3514059	6.35 × 10^−3^
IL-10	−0.3742493	3.50 × 10^−3^
variance explained: 12.24%		

For PCA2 IL-1β, IL-6, IFNγ and CXCL-1 loaded positively, whereas IL-4, IL-5 and IL-10 loaded negatively onto this component, which explained 12.24% of the variance. No significant effects between parity and age groups were demonstrated in PCA2 (all *p* > 0.191; figure 6*b*). Therefore, cytokine levels in the hippocampus appear to be quite stable, and unaffected by parity and age.

### 3.5. Parity transiently alters the tryptophan–kynurenine pathway

**Figure 7.**
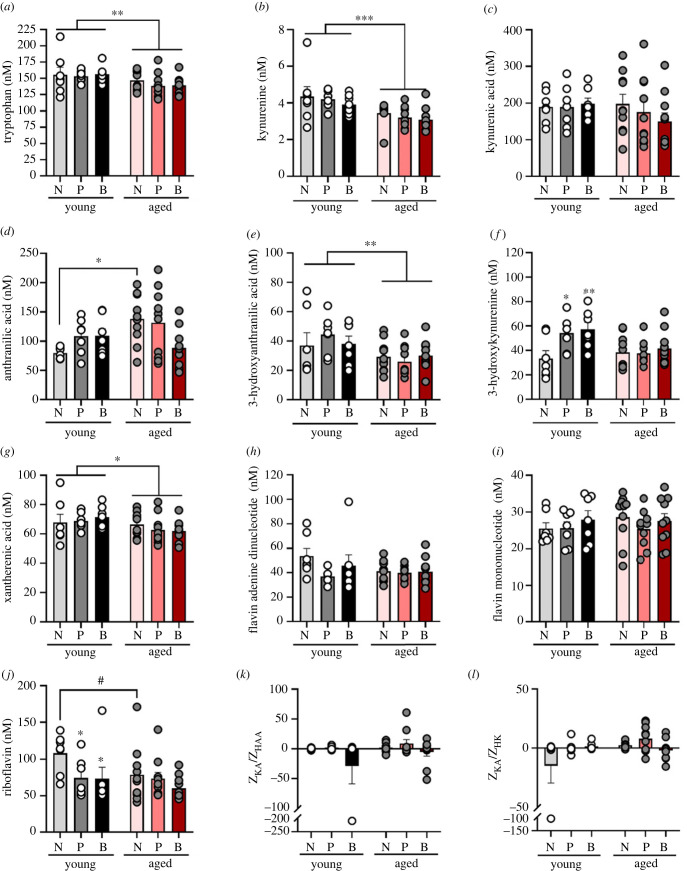
Parity alters the tryptophan–kynurenine pathway in young adult, but not aged females. Plasma levels of tryptophan (*a*), kynurenine (*b*), kynurenic acid (*c*), anthranilic acid (*d*), 3-hydroxyanthranilic acid (*e*), 3-hydroxykynurenine (*f*), xantherenic acid (*g*), flavin adenine dinucleotide (*h*), flavin mononucleotide (*i*) and riboflavin (*j*). Ratios of *Z*-scores for neuroprotective plasma metabolite kynurenic acid (KA) and neurotoxic 3-hydroxy anthranilic acid (HAA) and 3-hydroxykynurenine (HK) are demonstrated in K and L, respectively. Data are presented in nanomolar and are expressed as mean ± s.e.m. *n* = 6–7 (young) and 9–10 (aged) per group. *p < 0.05, **p < 0.01, ***p < 0.001, ****p < 0.0001, #*p* = 0.054.

The TRP–KYN pathway is stimulated by proinflammatory cytokines and is associated with several neurological diseases, such as neurodegenerative disease [56]. Tryptophan, KYN, XA and 3-HAA levels in plasma, were significantly reduced with age (main effect of age: *F*_1, 45_ = 7.298, *p* = 0.010, $\eta_{p}^{2}=0.140$; figure 7*a* [tryptophan]; *F*_1, 43_ = 20.661, *p* = 0.0004, $\eta_{p}^{2}=0.325$; figure 7*b* [KYN]; *F*_1, 45_ = 18.061, *p* = 0.0001, $\eta_{p}^{2}=0.286$; figure 7*e* [3-HAA]; *F*_1, 45_ = 4.567, *p* = 0.038, $\eta_{p}^{2}=0.092$; figure 7*g* [XA]). For 3-HK there was a significant interaction between parity and age (*F*_2, 45_ = 3.213, *p* = 0.050, $\eta_{p}^{2}=0.125$; figure 7*f*). Post-hoc analysis revealed that young adult nulliparous females had lower levels of 3-HK than both young primiparous (*p* = 0.030, Cohen's *d* = 1.337) and biparous (*p* = 0.014, Cohen's *d* = 1.457) females, but this difference was no longer present at middle age (*p* > 0.66).

For the metabolite AA, there was a significant interaction between parity and age (*F*_2, 45_ = 4.143, *p* = 0.022, $\eta_{p}^{2}=0.155$; figure 7*d*). Post-hoc analysis revealed that nulliparous aged females had significantly higher levels of AA than young adult nulliparous females (*p* = 0.045, Cohen's *d* = 1.897).

Next, we investigated the levels of the coenzyme FAD, which promotes the neurotoxic arm in the TRP–KYN pathway. There were no significant differences in FAD levels between age or parity groups (all *p* > 0.140; figure 7*h*). FMN was also not affected by age and/or parity (all *p* > 0.526; figure 7*i*).

Several key enzymes in the TRP–KYN pathway also require riboflavin (vitamin B2) as a cofactor [57], and riboflavin is an important vitamin during pregnancy that's heavily used during both pregnancy and postpartum. Young adult nulliparous females had significantly higher levels of riboflavin than the two young adult parous groups (primiparity: *p* = 0.041, Cohen's *d* = 1.272; biparity: *p* = 0.037, Cohen's *d* = 0.999), but there were no significant differences in the middle-aged group (parity by age interaction effect: *F*_2, 42_ = 4.579, *p* = 0.016, $\eta_{p}^{2}=0.179$; figure 7*j*). There were also significant main effects of parity (*F*_2, 42_ = 8.528, *p* = 0.0007, $\eta_{p}^{2}=0.289$) and age (*F*_1, 42_ = 6.848, *p* = 0.012, $\eta_{p}^{2}=0.140$).

Assessing a neuroprotective versus neurotoxic ratio of KA and 3-HAA, factors of the TRP–KYN pathway, did not reveal any significant main effects of parity, age, nor an interaction between the two (all *p's* > 0.101; figure 7*k*). Neither were there differences between groups when assessing the ratio of KA and HL (all *p's* > 0.426; figure 7*l*).

**Figure 8.**
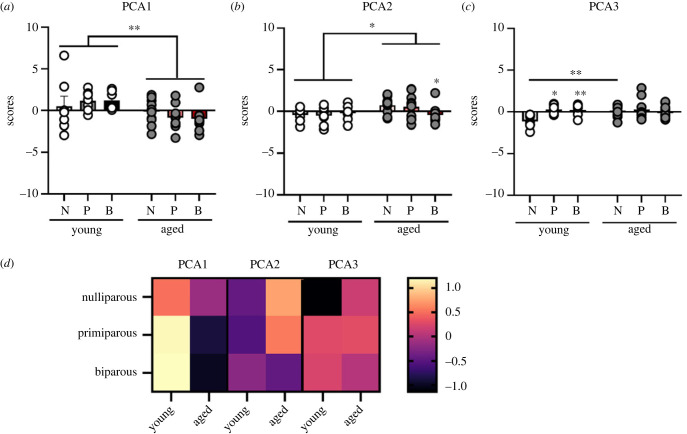
PCA for tryptophan–kynurenine metabolites. Loadings of scores for each subject onto PCA 1 (*a*), 2 (*b*) and 3 (*c*). Heat map displaying average loading of each parity group on each compartment (*d*). Data are expressed as mean ± s.e.m. *n* = 7 (young) and 10 (aged) per group. *p < 0.05, **p < 0.01. N, nulliparous; P, primiparous; B, biparous.

We also analysed the TRP–KYN metabolites using PCA. Component loading are displayed in table 2. Results from the PCA analysis yielded three principal components, which together explained 81.11% of the total variance. TRP, XA, KYN, 3-HK, 3-HAA and KA loaded positively onto PCA 1 which explained 49.97% of the variance (figure 8*a*). In PCA1, middle-aged females had lower scores than young adult females (main effect of age: *F*_1, 45_ = 10.622, *p* = 0.002, $\eta_{p}^{2}=0.191$) but no other significant effects (all *p* > 0.379).

**Table 2.** Component loadings of the plasma metabolite variance of components identified using PCA.

metabolic factor	correlation	*p*-value
PCA1		
tryptophan	0.023276	5.34 × 10^−22^
xanthurenic acid	0.9222197	7.38 × 10^−22^
kynurenine	0.8381485	1.69 × 10^−14^
3-hydroxykynurenine	0.6823298	3.50 × 10^−8^
3-hydroxyanthranilic acid	0.6238258	1.01 × 10^−6^
kynurenic acid	0.4251385	1.87 × 10^−3^
variance explained: 49.97%		
PCA2		
anthranilic acid	0.7237361	1.97 × 10^−9^
kynurenic acid	0.6562456	1.71 × 10^−7^
3-hydroxyanthranilic acid	−0.5331410	5.62 × 10^−5^
variance explained: 19.49%		
PCA3		
anthranilic acid	0.5492708	2.99 × 10^−5^
3-hydroxykynurenine	0.5209544	8.88 × 10^−5^
variance explained: 11.65%		

For PCA2 AA and KA loaded positively, whereas 3-HAA loaded negatively onto this component, which explained 19.49% of the variance. Age resulted in higher scores at middle age, compared with in younger females (main effect of age: *F*_1, 45_ = 4.735, *p* = 0.035, $\eta_{p}^{2}=0.095$; figure 8*b*). There were no other significant effects (all *p* *>* 0.128).

Lastly, AA and 3-HK, loaded positively to PCA3 which explained 11.65% of the variance (figure 8*c*). In PCA3 there is a significant interaction between parity and age (interaction: *F*_2, 45_ = 3.849, *p* = 0.029, $\eta_{p}^{2}=0.146$). Post-hoc analysis revealed that nulliparous females displayed significantly lower scores than primiparous (*p* = 0.010, Cohen's *d* = 2.133) and biparous (*p* = 0.009, Cohen's *d* = 1.866) females in the young adult age group. In addition, young adult nulliparous females had lower scores than middle age nulliparous females (*p* = 0.008, Cohen's *d* = 1.733) but this was not seen in the parous groups (all *p* > 0.878). There was no significant effect of age (*p* = 0.124).

Taken together, TRP–KYN metabolites were mostly affected by age, with few differences between parity groups which were present only in younger animals and dissipated with time.

### 3.6. Vitamin B6 levels are reduced with age

**Figure 9.**
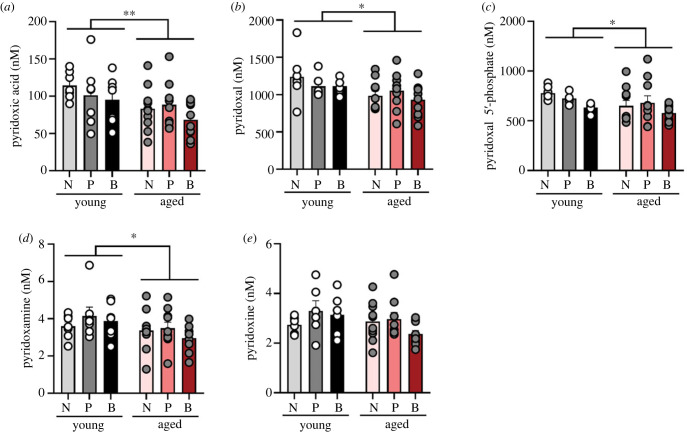
Levels of vitamin B6 coenzymes in plasma are reduced with age, regardless of parity. Levels of vitamin B6 coenzymes pyridoxic acid (*a*), pyridoxal (*b*), pyridoxal 5*′*-phostphate (*c*) and pyridoxamine (*d*) were reduced with age, but not or pyridoxine (*e*). Data are presented in nanomolar and expressed as mean ± s.e.m. *n* = 7 (young) and 10 (aged) per group. *p < 0.05, **p < 0.01.

The TRP–KYN pathway is sensitive to vitamin B6 availability, in which two of the key enzymes kynurenine aminotransferase and kynureninase require the active form of B6, pyridoxal 5′phosphate. Vitamin B6 is required for many processes during pregnancy, and vitamin B6 deficiency is common during this period [46]. Therefore, we measured vitamin B6 and its active coenzyme forms. The levels of pyridoxic acid, pyridoxal, pyridoxal 5′-phosphate and pyridoxamine were significantly reduced with age (main effect of age: *F*_1, 45_ = 7.768, *p* = 0.008, $\eta_{p}^{2}=0.147$, figure 9*a* [pyridoxic acid]; *F*_1, 43_ = 6.563, *p* = 0.014, $\eta_{p}^{2}=0.132$, figure 9*b* [pyridoxal]; *F*_1, 45_ = 6.619, *p* = 0.013, $\eta_{p}^{2}=0.128$, figure 9*c* [pyridoxal 5′-phosphate]; *F*_1, 45_ = 4.710, *p* = 0.035, $\eta_{p}^{2}=0.095$, figure 9*d* [pyridoxamine]), but there were no other significant main or interaction effects (all *p* *>* 0.235). For pyridoxine, neither the main effect of interaction, parity nor age were significant (*p* > 0.135, figure 9*e*).

## 4. Discussion

Pregnancy and the postpartum involves vast physiological changes, including effects on the central nervous system. Here we conduct a broad survey of cellular and molecular signatures of motherhood in the adult and ageing hippocampus. We examined the effects of primiparity and biparity on central and peripheral inflammation, metabolomics and synaptic plasticity after parturition and weaning in the short term (postpartum day 30) and at middle age (postpartum day 240). We found that parity results in short- and long-term changes to hippocampal synaptic protein PSD-95, cell signalling and inflammatory signalling levels, and the levels of certain inflammation-associated metabolites of the TRP–KYN pathway in plasma. The amount of parity was also important for certain outcomes as biparity had greater effects on inflammatory-related signalling (cell signalling proteins and microglia) in the hippocampus. Lastly, parity prevented the age-related decline in stem cells observed in nulliparous rats.

### 4.1. Parity regulated aspects of neuroplasticity in the short term and in the long term

In the current study, we found that previous parity increased hippocampal levels of PSD-95, pp70S6K and pS6RP, effects that were detected at postpartum day 30 and at middle age. Notably, effects on cell signalling phosphoproteins were only seen in biparous animals, highlighting the importance of considering amount of parity. PSD-95 is a very abundant scaffold protein in the PSD fraction, and its role is to organize the PSD by anchoring mostly glutamate channels and receptors [58,59]. Both phosphoproteins that were increased with biparity play important roles in synaptic plasticity. Furthermore, elevated S6RP phosphorylation is associated with increased synaptic plasticity in hippocampal, cortical and striatal neurons [60–65]. Pp70S6K, a protein in the protein kinase B (Akt) pathway, plays an important role in synaptic plasticity [66]. Thus, overall, we find that proteins related to synaptic plasticity are upregulated with parity in the hippocampus.

Our findings are consistent with previous work demonstrating increases in the synaptic proteins, spinophilin and synaptophysin, in the hippocampus or whole brain of multiparous rodents (mice and rats) in middle age [67,68]. Parity increases dendritic spine density in the CA1 region in late pregnancy, early postpartum (in lactating females; 5–6 days postpartum) and in older age (18 month) [69,70]. However, primiparity reduces CA3 and CA1 dendritic number and length in the late postpartum compared with biparous or nulliparous female rats [29]. In middle and older age, previous parity increases CA1 long-term potentiation [71], CA1 dendritic length [69], and the synaptic proteins spinophilin [68] and synaptophysin [67] in rodents. Together with the results of the current study, this indicates that increases in synaptic proteins are a feature of parenthood that is related to the amount of reproductive experience (primiparous, biparous, multiparous). Although the functional implications are not clear, studies indicate that synaptic protein levels are associated with cognitive ability in males and females [72,73]. Our findings are therefore consistent with previous studies reporting improvements in spatial learning and memory in parous females at the same time points (30 days postpartum and middle age) [21,67,74–76]. Furthermore, improvements in reference and working memory were reported 15 days after weaning in primiparous females, compared to age-matched females [77]. Maternal changes to synaptogenesis and cognition are similar to environmental enrichment in rodents, suggesting that the maternal experience is stimulating and protective for brain health [55]. In humans, de Lange and colleagues demonstrated that childbirth was associated with less apparent brain ageing, including in the hippocampus [24,78,79]. Thus, parity is associated with increases in synaptic proteins which may indicate improved memory and reduced brain ageing, although more research is necessary to establish this connection.

### 4.2. Biparity was associated with greater density of Iba-1+ in the dentate gyrus in females

In the present study, biparity resulted in a lasting increase in Iba-1+ cell density in the dentate gyrus, regardless of age when compared to nulliparous females. Although studies investigating age-related changes in microglia have produced varying results, this may be due to inconsistent inclusion of hippocampal subregions [80], sex and parity (due to the use of retired breeders). One previous study including females found an age-related increase in microglia in the dentate gyrus which was not present between young and aged males [81]. An age-dependent increase in Iba-1+ density was also reported in the dentate gyrus of primiparous, but not nulliparous, females [1].

Iba-1 is cytoplasmic marker used to identify microglia in mammalian species [82]. It is important to note that Iba-1 is not a specific marker to microglia, as it labels all subpopulations of cells of the macrophage lineage [83]. Therefore, while the major populations of macrophages in the brain are microglia, we cannot exclude that other populations of macrophages were included in the analysis.

Future work should examine cytokine signalling within subregions of the hippocampus and in other areas in which parity-related changes have been reported, for example, the amygdala, substantia nigra, hypothalamus and prefrontal cortex [5,24].

### 4.3. Parity prevented the age-related decline in neural stem cells in the hippocampus

Neural stem cells were reduced with age, as expected [84], but only in the nulliparous group. The same ageing effects were not seen in parous groups, indicating that previous parity may mitigate age-dependent declines in neural stem cells. These findings may explain the greater levels of neurogenesis in the hippocampus of parous rats in middle-age compared to nulliparous rats [1,26,74]. Further studies should be conducted to determine whether these neural stem cells are more likely to be dividing in middle aged parous compared with nulliparous rats.

### 4.4. Parity transiently alters the tryptophan–kynurenine pathway in plasma

The TRP–KYN pathway interacts with the immune system and its enzymes are expressed in many immune cells, including microglia and macrophages [85]. Only two plasma molecules differed between parity groups postpartum: 3-HK and riboflavin, also known as vitamin B2. 3-HK is increased during pregnancy, and we found levels of this metabolite remained significantly elevated at 30 days postpartum in both primiparous and biparous females, but there were no differences between parous groups in middle age. Vitamin B2 levels were significantly lower in primiparous and biparous females at 30 days postpartum, compared to age-matched nulliparous females, but these differences were no longer present at middle age. The risk of vitamin B2 deficiency is highest during the third trimester and lactation [86], and our results suggest that lower levels can persist in rats at least a week after the pups have been weaned. Although KYN, KA and XA are commonly elevated during pregnancy, we found no differences in the levels of these kynurenines at 30 days postpartum, indicating that their levels were not affected by the reduction of riboflavin in parous females. Not surprisingly, the most prominent changes to TRP–KYN metabolite levels were by age, as plasma levels of TRP, 3-HAA and XA were reduced at middle age, compared to in young adult females, consistent with other findings [87–89]. Reductions in TRP and XA have previously been linked to an increased risk of cognitive impairment [90]. However, in contrast to our results, KYN has previously been found to increase with age [91,92].

Levels of AA, the third immediate downstream KYN metabolite, were increased with age in plasma, but only in nulliparous females. In addition, plasma HK was increased 30 days postpartum in primiparous and biparous females, compared to nulliparous females. An increase in this metabolite has also been reported in humans around 40 days postpartum [93].

We found reduced levels of plasma pyridoxal 5′-phosphate with age. Age-related reductions were also found for other forms of vitamin B6 in plasma, including pyridoxic acid, pyridoxal and pyridoxamine. These findings are in line with other study showing lower levels of plasma pyridoxal 5′ phosphate in older adults [94,95]. However, parity did not alter the levels of any form of vitamin B6 postpartum or at middle age.

### 4.5. Reproductive experience influences brain ageing

Intriguingly, some effects of parity were only noted in the biparous group. Biparity increased levels of hippocampal cell signalling phosphoproteins, plasma metabolites and microglia in the dentate gyrus. Other groups have found that the amount of parity influences cognition, neuroplasticity, brain ageing, and potentially risk for ageing-related disorders [29,78,96]. In the short-term after parturition, biparity was associated with greater survival of new neurons across the postpartum [29]. During gestation and early postpartum, primigravid individuals outperform multiparous individuals on a verbal memory test [96]. Amount of parity also influences cognition and hormone levels, and potentially dementia, in humans. Those with 2–3 children demonstrated improvements in visual or verbal memory at middle age, compared to those with 0–1 children [25,73]. Some studies have found that Alzheimer's neuropathology is increased with parity [31], and that risk for Alzheimer's disease is increased with three or more pregnancies, but this may depend on country [97]. These differences between first and subsequent pregnancies may arise from differences in requirements of the mother. Levels of estrogens and progesterone are lower across the menstrual cycle after the first pregnancy, thus providing a different hormonal milieu for the second pregnancy [98] and lower levels of oestrogens, α-fetoprotein, human chorionic gonadotropin and prolactin are reported in subsequent pregnancies [98,99]. Thus, the observed differences between primiparous and biparous females may be attributed to various factors, including the cumulative effects of reproductive experiences, hormonal changes and the unique physiological adaptations associated with subsequent pregnancies.

Here, we report that ageing decreased neural stem cells in the dentate gyrus, and plasma AA, 3-HK and riboflavin, of nulliparous but not parous rats. This may be related to findings indicating less evident brain ageing in humans with childbirths, in the hippocampus as well as other regions [24]. Lastly, a new review by Orchard and colleagues indicates that reproductive experience may result in similar neural and cognitive benefits as environmental enrichment in rodents, as the continual adjustments and changing environments that we have to adapt to during a child's development result in sensory, cognitive, motor and social stimulation [55]. These effects can stimulate synaptogenesis [100,101] and may be protective against brain ageing [102].

## 5. Conclusion

We found that parity has a significant impact on a variety of neuroplastic markers, long after parturition, including some effects that lasted into middle age. Given that parity is associated with different disease risk for stroke, diabetes, and Alzheimer's disease, it is important to understand how the brain may change with parity and age. In our study, we found that parity increased hippocampal PSD-95 regardless of amount of parity, and that with biparity phosphoproteins related to synaptic plasticity, pp70S6K and S6RP, were increased in the hippocampus. Although neural stem cell density decreased in the hippocampus of nulliparous rats, this same profile was not seen in parous animals with ageing. Although biparous mothers had a higher level of microglia in the dentate gyrus than nulliparous females we did not see any robust parity-based changes in circulating or central inflammatory cytokine levels across the entire hippocampus. Future work could examine a more regional approach to inflammatory cytokines, in addition to immune-related changes in other brain regions. In addition, future work should examine implications of greater levels of parity, as grand parity (more than four pregnancies) is suggested to impair cognition and increase the risk of dementia later in life [103]. The TRP–KYN system was mainly affected by age, with a reduction in the levels of several key metabolites of this pathway at middle age in plasma. Although some parity related changes were found in TRP–KYN metabolites, these were limited to young adult females and did not persist until middle age. Therefore, parity alters the trajectory of brain ageing for certain parameters, whereas others were affected specifically by the presence of previous parity or age solely. By discerning the distinct contributions of parity to the ageing process, we can unravel the complex mechanisms underlying disease progression, ultimately enabling more effective preventive and therapeutic strategies.

## Data Availability

The datasets supporting this article have been uploaded as part of the electronic supplementary material [104].
